# Supplementary material for: A meta-analysis of structural MRI studies of the brain in systemic lupus erythematosus (SLE)
Source: Clin Rheumatol. 2022 Dec 19;42(2):319–26. doi: 10.1007/s10067-022-06482-8 (PMC9873736; doi:10.1007/s10067-022-06482-8)
Supplement: Supplementary file 1 — Supplementary file1 (DOCX 110 KB) [file 10067_2022_6482_MOESM1_ESM.docx]

**Supplementary Material for Cox et al. “A meta-analysis of structural MRI studies in systemic lupus erythematosus (SLE)”**

Contents

1. Search strategy for literature review and meta-analysis
2. PRISMA inclusion flow chart
3. Supplementary Tables
4. Search strategy for literature review and meta-analysis

Search Terms:

((Systemic Lupus Erythematosus) OR (SLE) OR (lupus) OR (neuropsychiatric systemic lupus erythematosus) OR (NPSLE) OR (lupus vasculitis) OR (rheumatoid arthritis) OR (ulcerative colitis)) AND (volume OR Hippocampus OR sulcus OR gyrus OR putamen OR caudate OR ventricle OR (prefrontal) OR parietal OR occipital OR cingulate OR amygdala OR (“white matter”) OR (“grey matter”) OR (“gray matter”) OR cortex) AND MRI

Database Used:

PubMed

Original Search Date:

18 November 2020

Updated Search Date:

03 March 2022

Restrictions:

- English language only
- Must include subjects with Systemic Lupus Erythematosus (SLE), Rheumatoid Arthritis (RA) or Ulcerative Colitis (UC)
- Have reported structural neuroimaging measures available
- Include a control arm in the study design
- All case studies/case series excluded

1. PRISMA inclusion flow chart


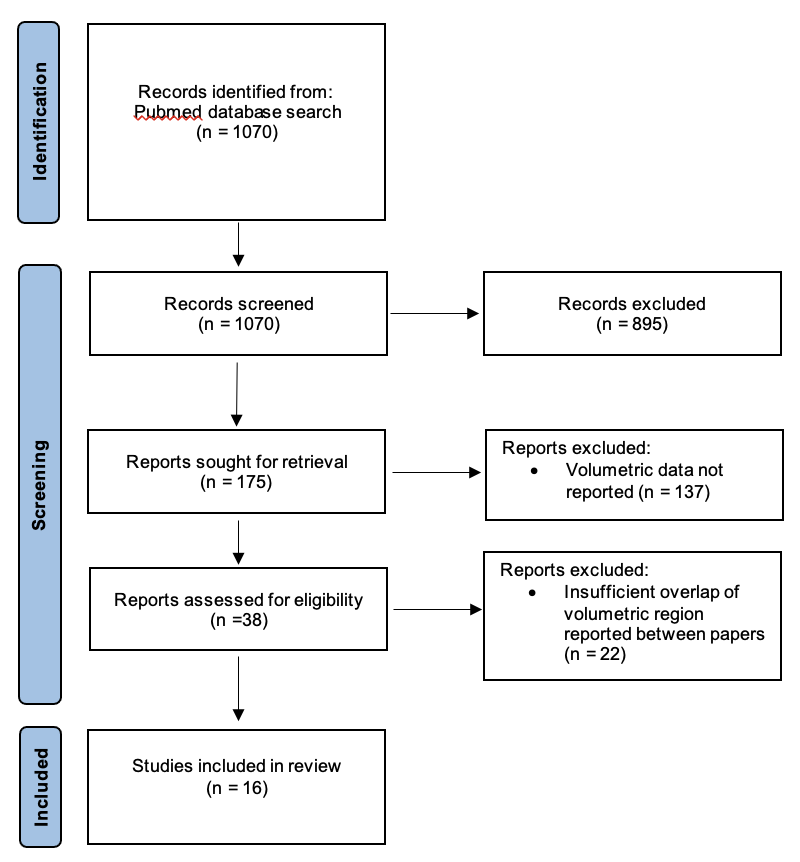


1. Supplementary Tables

Table 2: Studies included in the SLE Meta-Analysis

| Study | No. of SLE Patients | No. of Controls | Brain Regions Reported | Measurement Method Used ^a^ |
| --- | --- | --- | --- | --- |
| Kozora et al, ^[28]^ 2005 | 7 | 7 | Hippocampus | Manual segmentation |
| Appenzeller et al, ^[29]^ 2006 | 107 | 40 | Hippocampus | Manual segmentation |
| Shapira-Lichter et al, ^[30]^ 2013 | 12 | 11 | Hippocampus | Manual segmentation |
| Lapa et al, ^[31]^ 2017 | 54 | 56 | Hippocampus | Manual segmentation |
| Cannerfelt et al, ^[22]^ 2018 | 27 | 25 | Hippocampus, Corpus Callosum | Freesurfer |
| Liu et al, ^[32]^ 2020 | 85 | 77 | Hippocampus | SPM |
| Kamintsky et al, ^[39]^ 2020 | 65 | 9 | Hippocampus | VolBrain |
| Appenzeller et al, ^[33]^ 2005 | 115 | 44 | Corpus Callosum | Neuroline |
| Lee et al, ^[23]^ 2015 | 12 | 22 | Corpus Callosum | SPM |
| Tamires Lapa et al, ^[13]^ 2016 | 76 | 66 | Corpus Callosum | Neuroline |
| Emmer et al, ^[34]^ 2006 | 21 | 12 | Total Gray Matter | SNIPER |
| Cagnoli et al, ^[35]^ 2012 | 18 | 18 | Total Gray Matter | SPM |
| Tjensvoll et al, ^[36]^ 2016 | 53 | 53 | Total Gray Matter | SPM |
| Liu et al, ^[24]^ 2018 | 89 | 84 | Total Gray Matter | SPM |
| Zivadinov et al, ^[37]^ 2013 | 26 | 36 | Total Gray Matter | SIENAX |
| Cesar et al, ^[38]^ 2015 | 23 | 43 | Total Gray Matter | SIENAX |

^a^ Acronyms for measurement methods: Statistical Parametric Mapping (SPM), Software for Neuro-Image Processing in Experimental Research (SNIPER), Structural Image Evaluation using Normalisation of Atrophy (SIENAX)
